# Rhinolithiasis: about an observation of a rare condition

**DOI:** 10.11604/pamj.2018.31.78.16570

**Published:** 2018-10-03

**Authors:** Jawad Lahma, Reda Hejjouji, Imane Azzam, Abdelilah Oujilal, Leila Essakalli

**Affiliations:** 1 ENT Department, Ibn Sina University Hospital, Mohammed V University, Rabat, Morocco

**Keywords:** Rhinolithiasis, nasal rhinorrhea, nasal foreign body, nasal endoscopy, rhinoliths

## Abstract

Rhinolithiasis is a rare condition often neglected or unknown that tends to disappear in developed countries and corresponds to a solid calcification by gradual deposition of calcareous salts around a central resorbable or non-resorbable foundation of varying shape and size. The most common symptom is a long-term unilateral purulent rhinorrhea and unilateral nasal obstruction. Nasal endoscopy and imaging are interesting for the positive diagnosis but especially to highlight the anatomical anomalies or related pathologies. Therapeutic management requires endonasal extraction of the rhinolith under general anesthesia. We report an observation of rhinolithiasis treated in our department associating a significant deformation of the nasal pyramid to osteolysis.

## Introduction

Rhinolithiasis is a rare condition often neglected or unknown that tends to disappear in developed countries and corresponds to a solid calcification by gradual deposition of calcareous salts around a central resorbable or non-resorbable foundation of varying shape and size [1]. Nasal endoscopy and imaging are interesting for the positive diagnosis but especially to highlight the anatomical anomalies or related pathologies. We report an observation of rhinolithiasis treated in our department associating a significant deformation of the nasal pyramid to osteolysis.

## Patient and observation

**Figure 1:**
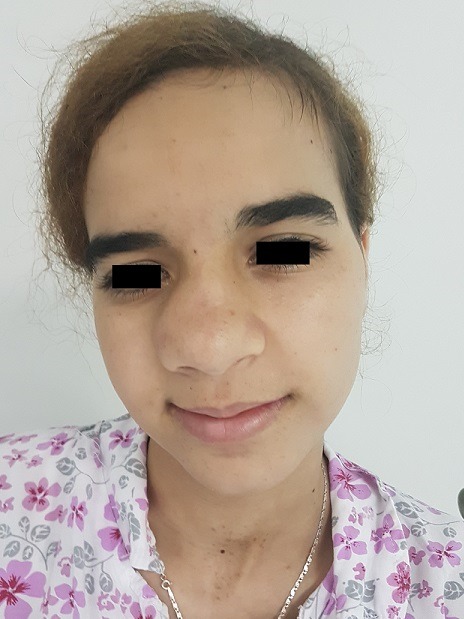
Deformation of the nasal pyramid on the right side

**Figure 2:**
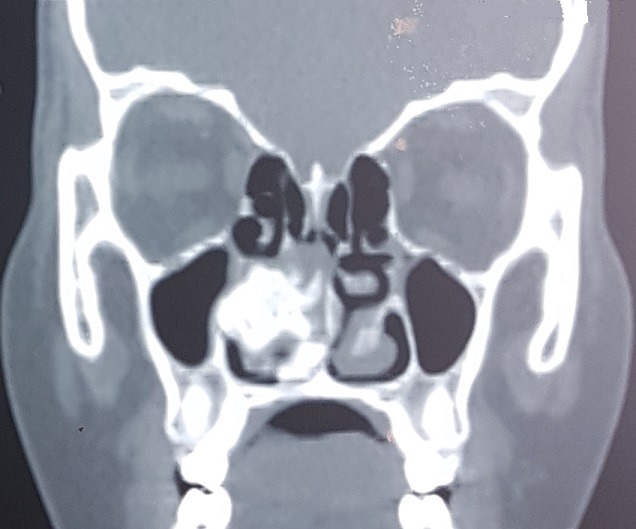
Coronal sinonasal computed tomography (CT) showing a high-density, calcified mass located between the inferior turbinate and nasal septum in the right nasal cavity with destruction of medial wall of maxillary sinus

**Figure 3:**
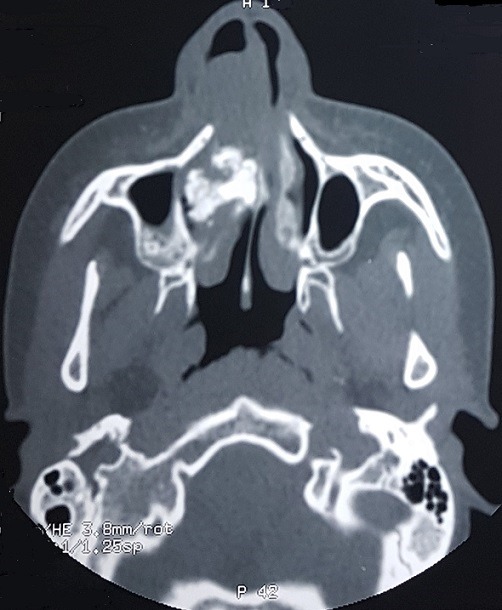
Axial sinonasal computed tomography (CT) showing rhinolite at the medium 1/3 of the right nasal fossa

**Figure 4:**
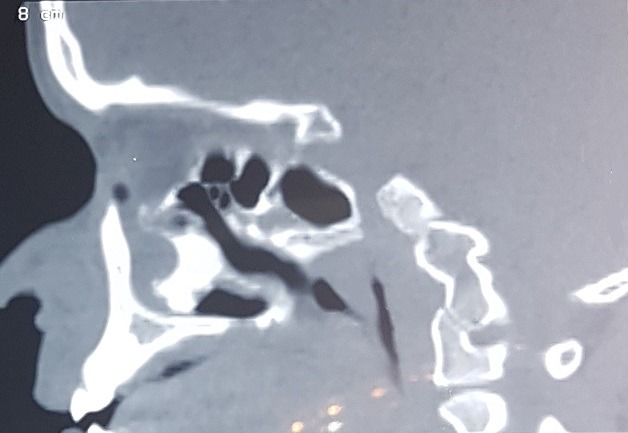
Sagittal sinonasal computed tomography (CT) showing incarceration of the rhinolite at the level of the inferior turbinate

**Figure 5:**
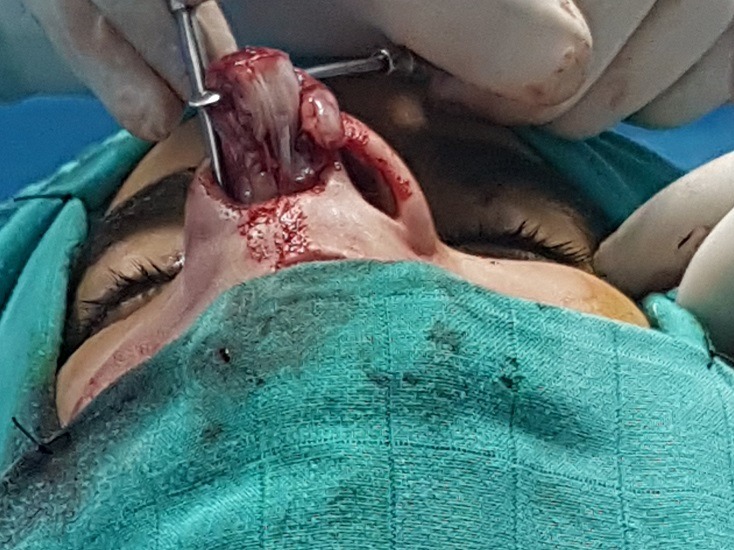
Extraction of the rhinolithe from back to front with a hook

**Figure 6:**
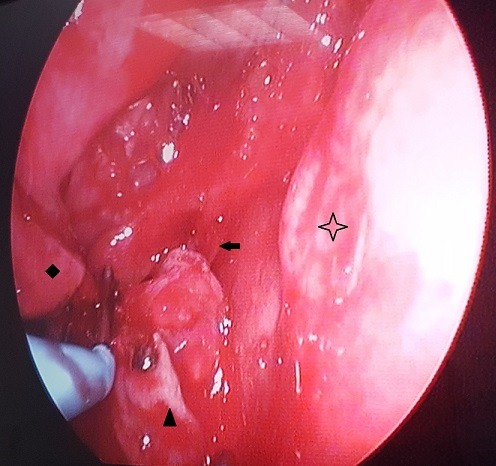
Endoscopic view of nasal cavity showing the posterior part of the rhinolite: tail of the inferior turbinate: choana: nasal septum

**Figure 7:**
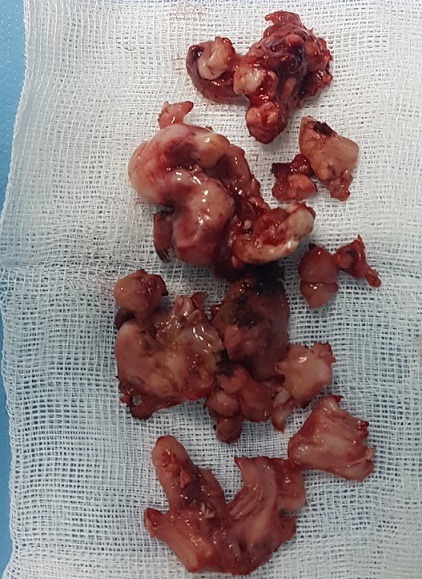
Image of extracted rhinolithiasis rounded by granulation and inflammatory tissue

Patient JD, 16 years old, without significant pathological antecedents, consults for a significant deformation of the nasal pyramid on the right side. The beginning of the symptomatology goes back to 4 years by the progressive installation of a right nasal obstruction associated with an anterior and posterior fetid chronic rhinorrhea unimpeded by well-conducted antibiotic treatment with occurrence of a progressive deformation of the ipsilateral nasal pyramid (Figure 1). The endonasal examination after aspiration of the purulent secretions showed an irregular granulomatous tissue mass at the level of the right nostril vestibule not allowing the introduction of the rigid optics due to its large volume and which is hard on palpatation with the stylet. The nasosinusal tomodensitometry examination showed 3 cm long axis calcium density opacity, with a hypodensity at its center, with destruction of medial wall of maxillary sinus which led us to make the diagnosis of rhinolithiasis (Figure 2, Figure 3, Figure 4 ). The extraction was carried out under general anesthesia under control of a rigid endoscope 0°, a hook having allowed the rhinolite to be brought from back to front (Figure 5) and a lower turbinectomy was performed due to its incarceration at the level of the inferior turbinate (Figure 6, Figure 7). Endoscopic verification showed complete emptiness of the right nasal fossa. The evolution was favorable after 6 months of decline.

## Discussion

Rhinolithiasis is a rarely published and uncommon entity; The first description of rhinolithiasis was reported by BARTHOLIN in 1654 [2]. In 1943 POLSON had collected 380 cases [3]. We can estimate at 800 the number of cases published in the literature [1]. They are rare in developed countries and they affect commonly young adult, but it could be seen at all ages [2]. Rhinoliths are often occurred in female patients [3, 4]. Pathogenically, we recognize the primitive rhinolithiases (endogenous) where the anatomical substratum consists of lysis debris and cellular desquamation associated with changes in the viscosity of nasal secretions potentiated by changes in the quality of the environment (pollution) [1]. The chemical composition of rhinolite is made of water (2.9 to 5.9%), magnesium phosphate (19.46%), calcium carbonate (20.69%), calcium phosphate (44%), 7%) and organic substances (13.2%) [1, 5]. Secondary (exogenous) rhinolithiases are formed from an unrecognized or neglected organic or non-organic foreign body (pearls, toy debris, ectopic teeth, surgical sponges) [5, 6]. The rhinoliths formation period varies from a few months to several years [1, 2]. Clinically, the symptomatology is dominated by unilateral chronic purulent rhinorrhea and nasal obstruction [4]. Furthermore other symptoms can be found as epistaxis, headache, cacosmia, anosmia and facial pain [1-4, 7]. However, rhinolithiasis may be asymptomatic and found incidentally on routine ENT examination, nasosinusal CT scan, dental radiographic films [4, 7, 8]. Rhinoscopy and Endoscopic nasal examinations allow to evoke the diagnosis by showing a yellow-gray spiculate mass of variable size and shape at the inter-septum turbinal space rounded by granulation and inflammatory tissue [7]. Two clinical characters confirm the diagnosis: the very hard appearance (stony consistency) and the sensation of crackling during the button-styled exploration [1, 9]. Endoscopy allows a lesional assessment: septal perforation [9], synechia, granuloma and reaction polyp. Nasosinusal computerized tomography (CT) findings is the exam of choice for the diagnosis of rhinolithiasis. It reveals a homogeneous high-density mass with irregular contours and a hypodense nidus in the central part of the lesion that sometimes may cause septal perforation, destruction of medial wall of maxillary sinus with recurrent sinusitis, palatal perforation, oral fistula and rarely osteomyelitis and epidural abscess [1, 7, 9-11]. This CT scan sometimes helps to recognize its origin (foreign body metallic, ectopic nasal tooth) [12]. This findings is also very useful in the choice of the process therapeutic approach (approach, prediction on the difficulties of extraction) and for differential diagnosis that include osteoma, calcified polyps, bone sequestration (syphilis, radiotherapy); osteosarcoma and chondrosarcoma [1]. The ideal therapeutic management is based on the extraction of rhinolite by natural routes under general anesthesia with or without fragmentation of rhinoliths [2, 13]. Extraction requires endoscopic approach with rigid optics that ensures good working conditions. The use of general anesthesia with orotracheal intubation is necessary in the child, the pusillanimous subject, in case of giant rhinolite or enclosed in the posterior part of the nasal fossae and if there are associated lesions (sinusitis, polyp, major hypertrophic rhinitis, mycosis). However the small rhinolith may be removed under local anesthesia [7]. Removal of the rhinolite by external surgical means by Caldwell -Luc approach or lateral rhinotomy incision is exceptionally indicated, particularly in the case of giant rhinolite associated with a giant turbino-septal malformation and in the event of massive granulomatous reaction including the rhinolite. Lithotripsy, although reported by some authors, is not a therapeutic standard [14]. After extraction the physico-chemical expertise of the rhinolite (Measurement, weighing, search for a central foreign body and Histo-biochemical study) is carried out [15]. Recurrences of rhinolithiasis are exceptional and outcomes are generally favorable [16].

## Conclusion

Cases of rhinolithiasis are seen rarely and have progressive installation over dozens of years. The most common symptom is a long-term unilateral purulent rhinorrhea and unilateral nasal obstruction. Nasal endoscopy and imaging data confirm the diagnosis. Therapeutic management requires endonasal extraction of the rhinolith under general anesthesia.

## Competing interests

The authors declare no conflict of interest.

## References

[1] Kharoubi S (2007). Revue générale sur les rhinolithiases: à propos de 20 cas. J Tun ORL..

[2] Eliachar I, Schalit M (1970). Rhinolithiasis. Report of eight cases. Arch Otolaryngol..

[3] Polson CJ (1943). On rhinoliths. J Laryngol Otol..

[4] Ozdemir S, Akbas Y, Görgülü O, Selçuk T, Sayar C (2010). Rhinolithiasis: review of 21 cases. Am J Rhinol Allergy..

[5] François M (2010). Corps étrangers des fosses nasales. Rhinolithiase. EMC - Oto-rhino-laryngologie..

[6] Janardhan N, Kumar S, Reddy R, Kumar C (2013). Rhinolithiasis due to supernumerary ectopic tooth: very rare case. Indian J Otolaryngol Head Neck Surg..

[7] Nadir Y, Atilla A, Murat S, Altan Y (2008). Rhinolithiasis: Clinical, radiological, and mineralogical Features. Am J Rhinol..

[8] Karli R, Ak M, Karli A (2012). A different placement of the stone; rhinolithiasis. Eur Rev Med Pharmacol Sci..

[9] Kharoubi S (1998). Rhinolithiasis associated with septal perforation: a case report. Acta Otorhinolaryngol Belg..

[10] Igoumenakis D, Athanasiou S, Mezitis M (2013). A bizarre cause of extensive oronasal fistula. J Craniofac Surg..

[11] Tmaca S, Belet N, Sensoy G, Belet U (2010). Rhinolithiasis: an unusual cause of sinusitis complicated with frontal osteomyelitis and epidural abscess. Turk J Pediatr..

[12] Kaushik V, Bhalla K, Pahade A (2004). Rhinolithiasis. Ear Nose Throat J..

[13] Aksungur EH, Binokay FB, Bicakci K, Apaydin D, Oguz M, Aydogan B (1999). A rhinolith which is mimicking a nasal benign tumor. Eur J Radiol..

[14] Mink A, Gati I, Szekely J (1991). Nasolith removal with ultrasound lithotripsy. HNO..

[15] Vink, BW-Van Hasselt P, Wormald R (2002). A case of rhinolithiasis in Botswana: a mineralogical, microscopic and chemical study. J Laryngol Otol..

[16] Dogan M, Dogan D, Düger C, Polat S, Muderris S (2012). Recurrent rhinolithiasis: a case report with review of the literature. West Indian Med J..

